# Can repair be an alternative to reconstruction in the management of acute anterior cruciate ligament rupture: A meta‐analysis of randomized controlled trials

**DOI:** 10.1002/jeo2.70235

**Published:** 2025-04-13

**Authors:** Ting Zhang, Xiaojin Ye, Qing Huang, Ke Zhou, Jin Li, Kaifeng Gan

**Affiliations:** ^1^ Department of Orthopedics The Affiliated LiHuiLi Hospital of Ningbo University Ningbo Zhejiang China; ^2^ Department of Orthopedics, The First Affiliated Hospital Zhejiang University School of Medicine Hangzhou China

**Keywords:** ACL injury, ACL repair, ACL reconstruction, meta‐analysis

## Abstract

**Purpose:**

To perform a meta‐analysis to compare the clinical outcomes and failure rate of anterior cruciate ligament (ACL) repair and ACL reconstruction in acute ACL rupture.

**Methods:**

Studies were searched on PubMed, Web of Science, and EMBASE for randomized controlled trials comparing ACL repair versus ACL reconstruction for ACL rupture. The bias risk was based on the Cochrane Handbook for Systematic Reviews of Interventions. Clinical outcomes included IKDC score, Lysholm score, Tegner score, anteroposterior (AP) knee laxity, and failure rate. The funnel plots were used to detect publication bias.

**Results:**

Six randomized controlled study (RCTs) were included in this meta‐analysis, involving a total of 478 patients. The mean follow‐up varied from 1 to 5 years. The mean age of patients was between 17 and 29.1 years, the mean time from injury to surgery was 13–39 days. We found no statistical differences between ACL repair and ACLR in IKDC score (0.11; 95% CI, −0.17 to 0.40; *p* = 0.440; *I* ^2^ = 56.8%), Lysholm score (0.16; 95% CI, −0.10 to 0.42; *p* = 0.214; *I* ^2^ = 28.8%), Tegner score (0.05; 95% CI, −0.23 to 0.34; *p* = 0.719; *I* ^2^ = 0.0%), AP knee laxity (0.05; 95% CI, −0.17 to 0.27; *p* = 0.636; *I* ^2^ = 0.0%), and failure rate (RR, 1.10; 95% CI, 0.70–1.72; *p* = 0.695; *I* ^2^ = 27.3%).

**Conclusion:**

ACL repair showed similar clinical outcomes compared with ACLR, and it could replace ACLR as an appropriate surgical method for acute proximal ACL rupture, but the indication and selection of patients are essential to be considered. Large numbers and more high‐quality studies are still needed in the future to verify our results.

**Level of Evidence:**

Level I.

**Abbreviations**
ACL: anterior cruciate ligament
ACLR: anterior cruciate ligament reconstruction
CI: confidence interval
IKDC: International Knee Documentation Committee
LOE: level of evidence
RCT: randomized controlled study

## INTRODUCTION

The anterior cruciate ligament (ACL) injury is prevalent, with an incidence of approximately 68.6 per 100,000 person‐years in the general population, particularly among athletes [20, 24]. ACL reconstruction (ACLR) has been considered the gold standard for treating ACL injuries, as it effectively restores knee stability and enhances functional outcomes. However, the results remain less than satisfactory, with revision rates reaching 10% due to complications such as long‐term poor stability and other adverse effects [17, 23, 25].

The first documented ACL repair was performed in 1895, and due to promising short‐term results, this technique gained widespread use until the 1980s. Unfortunately, medium‐ and long‐term outcomes revealed high failure rates and suboptimal clinical results [7], leading surgeons to abandon ACL repair by the end of the 20th century. Recently, however, primary ACL repair for proximal or midsubstance tears has garnered renewed interest, thanks to advancements in arthroscopic techniques and rehabilitation strategies, which address some of the complications associated with ACLR [29]. Theoretically, preserving the native ACL could maintain proprioception, and several studies have suggested that ACL repair for Sherman type I and II injuries can yield satisfactory clinical outcomes [5, 29]. Moreover, numerous clinical studies evaluating various repair techniques—such as suture anchor repair (fixing the ACL to the femoral footprint with anchors), bridge‐enhanced repair (utilizing a biological scaffold), dynamic intraligamentary stabilization (involving the implantation of a dynamic screwspring mechanism in the tibia), and internal brace ligament augmentation (adding an internal brace to the initial fixation for enhanced strength)—have reported encouraging results, with some even showing superiority over ACLR [6, 16, 18]. Nevertheless, the prioritization of ACL repair remains contentious. Some systematic reviews and meta‐analyses have indicated that ACL repair is associated with higher failure rates compared to ACLR [19, 27]. Conversely, other reviews suggest that ACL repair can achieve reasonable outcomes when compared to ACLR [1, 33]. Importantly, many previous meta‐analyses included studies that did not differentiate between tear types or account for the time interval from injury to surgery. It has been noted that early repairs are more likely to succeed than delayed interventions, and that repairs of type I and II injuries are more suitable than those of type III and IV injuries [30]. Additionally, these meta‐analyses often exhibited lower quality and high risk of bias. To address these gaps, we conducted a meta‐analysis of selected randomized controlled trials (RCTs) to provide a more reliable and rigorous conclusion regarding the viability of ACL repair as an alternative to ACLR for acute ACL injuries. The objective of this meta‐analysis was to compare the clinical outcomes and failure rates between ACL repair and ACLR. We hypothesized that ACL repair for acute ACL injuries would yield favorable clinical outcomes and lower failure rates compared to ACLR, potentially establishing it as a viable alternative.

## METHODS

### Literature search

The PRISMA (Preferred Reporting Items for Systematic Reviews and Meta‐Analyses) guidelines were used to design our meta‐analysis. Two independent reviewers performed the literature search based on the Preferred Reporting Items for Systematic Reviews and Meta‐Analysis guidelines [22]. A search of literature was conducted from January 2000 to July 2024 by using the electronic databases Web of Science, PubMed and EMBASE. The search strategies employed a combination of entry words and Medical Subject Headings (MeSH) terms with the following inputs: “anterior cruciate ligament” combined with the keywords “repair” or “reattachment” or “reinsertion” and “reconstruction” or “replacement,” with limitation to English‐language only.

### Inclusion and exclusion criteria

The retrieved articles were initially screened for relevance by the title and abstract. The eligibility criteria were (1) RCTs with Level I and II evidence, comparing primary ACL repair with ACLR; (2) the interval from injury to surgery was within 6 weeks; (3) adequate statistical power to defect differences with 95% confidence intervals (CIs); (4) a minimum of 1‐year; (5) clinical outcomes were reported; and (6) arthroscopy was used. Exclusion criteria were (1) non‐RCTs, nonclinical studies; case control studies, case series, retrospective cohort study, case reports or reviews; (2) a history of previous injury, revision surgery, severe or irreparable meniscal injury, or multiple ligament injury; (3) inadequate follow‐up; and (4) studies with only abstract available.

### Quality assessment

The entire text was obtained and further reviewed for its quality according to the Cochrane Handbook for Systematic Reviews of Interventions [4]. Two independent observers assessed potential biases including selection bias, performance bias, attrition bias, detection bias, and reporting bias. Additionally, the level of evidence (LOE) was determined for each eligible study (as given at http://handbook.cochrane.org/).

### Data extraction

Data were extracted independently from the remaining high‐quality studies that were all LOE I and II, including authors, year of publication, surgical procedure, sample size, age, location of ligament rupture, time from injury to surgery, clinical outcomes, and failure rate. Any discrepancies between the extracted data were resolved by consensus.

### Statistical analysis

Statistical analysis was performed using Review Manager 5.3 (Cochrane Collaboration, Nordic Cochrane Centre, Copenhagen, Denmark). Dichotomous variables were analyzed using relative risks, and continuous variables were assessed using the mean difference. We reported both with 95% CIs, and a *p*‐value < 0.05 was considered to be statistically significant. Heterogeneity between studies was evaluated by the Q statistic (significance level at *p* < 0.01) and the *I* ^2^ statistic (significance level at *I* ^2^ > 50%). A random‐effect method was adopted for the pooling of results if the Q or *I* ^2^ value was statistically significant; otherwise, a fixed‐effects model was used. Funnel plots and Egger test were used to detect publication bias.

## RESULTS

### Characteristics of the selected studies

**Figure 1:**
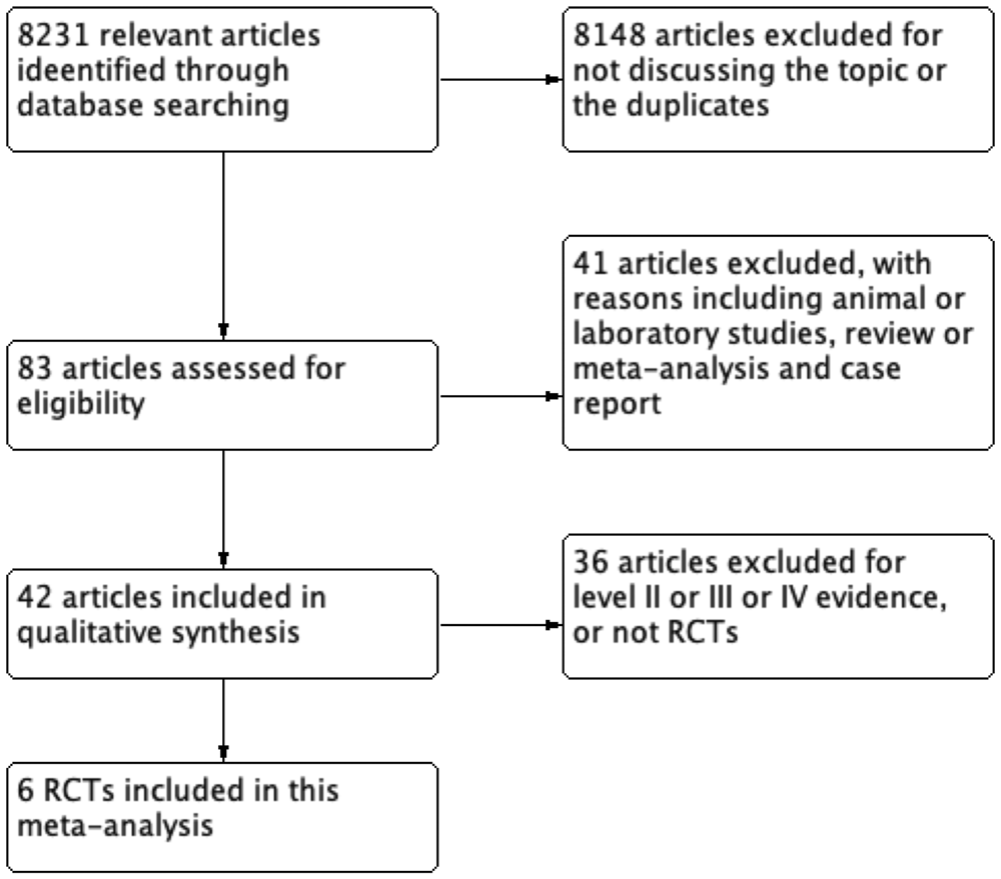
Trial flow. RCT, randomized controlled trial.

A summary of the study selection process was performed in Figure 1. The literature search identified 8231 relevant articles. From these, a total of 8148 citations were excluded as they did not meet the eligibility criteria. Following a review of the full text of 83 remaining articles, six studies [3, 8, 11, 15, 21, 26] were included in the quantitative analysis. All included studies were randomized controlled trials, with 478 participants enrolled overall (270 for ACL repair and 208 for ACLR). The quality of involved studies was assessed for risk of bias, including selection, performance, attrition, detection and reporting bias (Table 1). These studies were all randomized controlled trials of level of evidence I. Two studies adopted double‐blind methods, and three studies adopted the single‐blind method. All studies did a qualified follow‐up, and few patients were lost during the course of study. The follow‐up ranged from at least 1 year to 5 years. The mean age of patients was between 17 and 29.1 years, the mean time from injury to surgery was 13 and 39 days. All studies involved patients with the clinical outcome scores, and failure rates were reported with at least 1‐year follow‐up. The characteristics of the included studies were summarized in Table 2.

**Table 1:** Quality analysis of selected studies.

Authors	Study design	LOE	Selection bias	Performance bias	Detection bias	Attrition bias	Reporting bias
Barnett et al. [3]	RCT	I	Low	Low	Low	Low	Low
Glasbrenner et al. [8]	RCT	I	Low	Unclear	Low	High	Low
Hoogeslag et al. [11]	RCT	I	Low	High	High	Low	Low
Murray et al. [21]	RCT	I	Low	Low	Low	Low	Low
Schliemann et al. [26]	RCT	I	Low	Unclear	Unclear	Low	Low
Kösters et al. [15]	RCT	I	Low	Unclear	Low	Low	Low

Abbreviations: LOE, level of evidence; RCT, randomized controlled trial.

**Table 2:** Characteristics of the included studies.

Authors	No. patients		Intervention		Age		Sex M/F		Follow‐up	Time from injury to surgery (day)	
	Repair	ACLR	Repair	ACLR	Repair	ACLR	Repair	ACLR		Repair	ACLR
Barnett et al. [3]	65	35	BEAR	ST	17 (16–20)	17 (15–23)	28/37	16/19	2 y	36 (29–42)	39 (33–43)
Glasbrenner et al. [8]	43	42	DIS	ST	28.7 ± 11.4	27.6 ± 10.6	25/18	31/11	5 y	14.5 ± 5.2	16.7 ± 7.3
Hoogeslag et al. [11]	24	24	DIS	ST	21 (10–27)	22 (19.3–25)	19/5	18/6	5 y	13 (12–16)	47 (42–71)
Murray et al. [21]	65	35	BEAR	ST	17 (16–20)	17 (15–23)	28/37	16.19	2 y	36 (29–42)	39 (33–43)
Schliemann et al. [26]	30	30	DIS	ST	28.2 ± 11.4	29.1 ± 12.0	15/15	22/8	1 y	15.2 ± 4.5	16.3 ± 5.0
Kösters et al. [15]	43	42	DIS	ST	28.7 ± 11.4	27.6 ± 10.6	15/18	31/11	2 y	14.5 ± 5.2	16.2 ± 7.3

Abbreviations: ACLR, anterior cruciate ligament reconstruction; BEAR, bridge‐enhanced ACL repair; DIS, dynamic intraligamentary stabilization; SST, semimembranosus and semitendinosus tendon.

### Clinical outcomes

**Figure 2:**
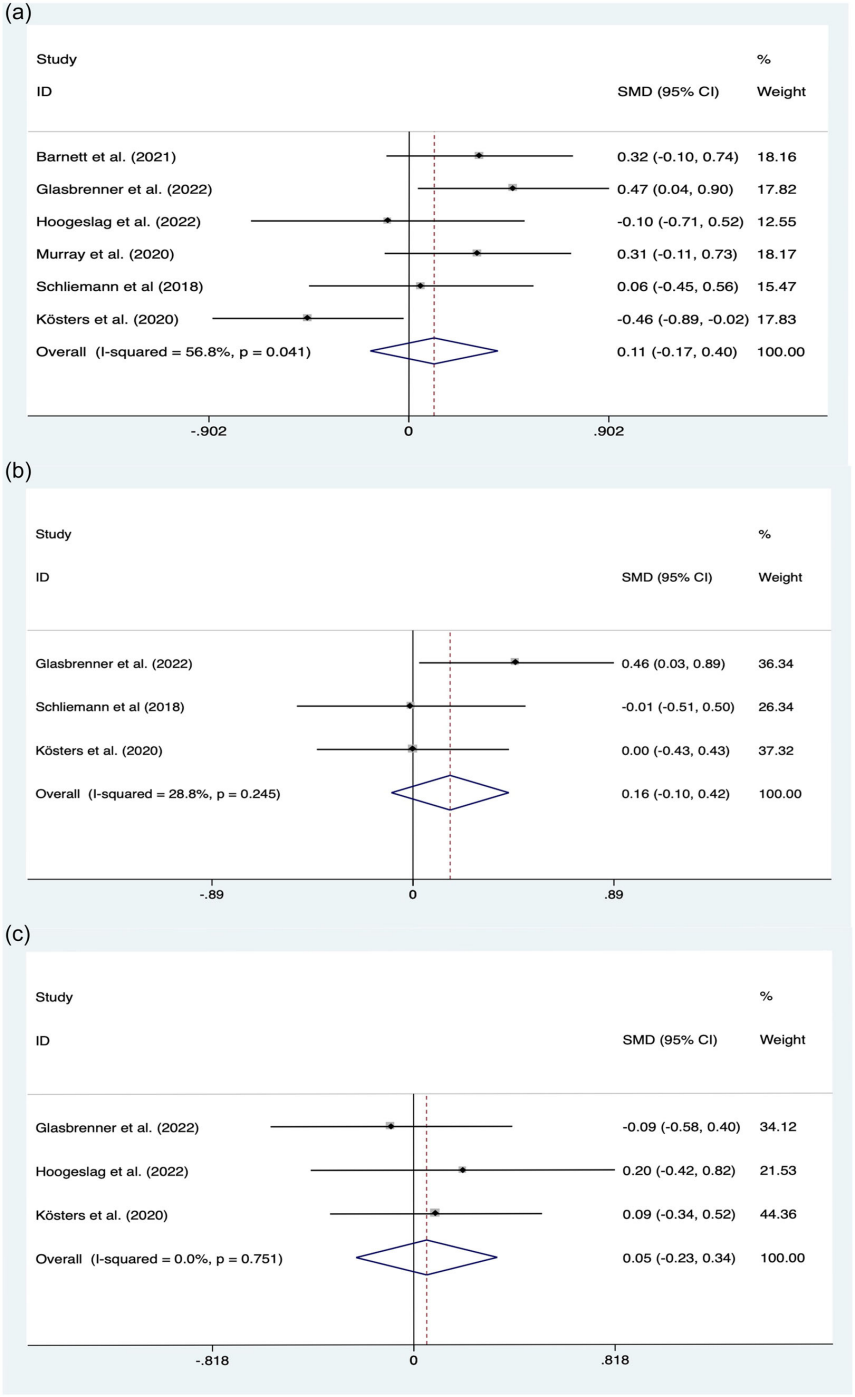
Meta‐analysis of clinical outcomes between ACL repair and ACLR. (a) IKDC score; (b) Lysholm score; (c) Tegner score. ACLR, anterior cruciate ligament reconstruction; IKDC, International Knee Documentation Committee.

Six studies, including 263 and 200 patients in ACL repair and ACLR groups, respectively, reported IKDC scores, and the results of pooled outcomes showed that the ACL repair was as good as ACLR (0.11; 95% CI, −0.17 to 0.40; *p* = 0.440; *I* ^2^ = 56.8%) (Figure 2a). Lysholm score was reported in three studies, and the results of meta‐analysis showed that ACL repair had a similar result compared with ACLR (0.16; 95% CI, −0.10 to 0.42; *p* = 0.214; *I* ^2^ = 28.8%) (Figure 2b). Besides, there was also no significant difference in Tegner scores between repair and ACLR groups (0.05; 95% CI, −0.23 to 0.34; *p* = 0.719; *I* ^2^ = 0.0%) (Figure 2c).

### Anterior posterior knee laxity

**Figure 3:**
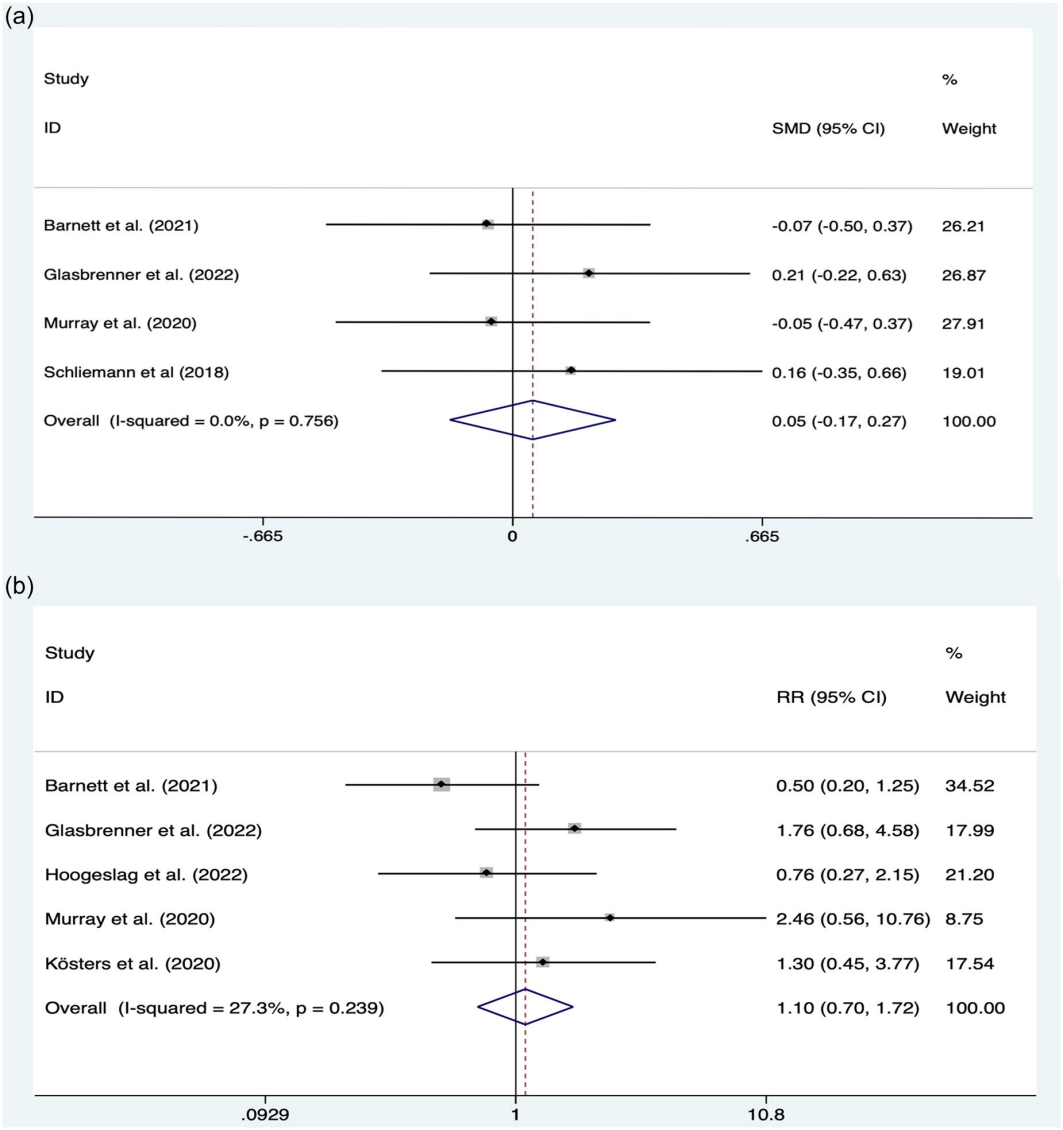
Meta‐analysis between ACL repair and ACLR. (a) AP knee laxity; (b) failure rate. ACLR, anterior cruciate ligament reconstruction; AP, anteroposterior.

Side‐to side difference in anterior tibial translation (∆ATT) was reported in 4 studies, comprising 193 and 138 patients in the repair and ACLR groups, respectively. The pooled outcomes showed that ∆ATT in ACLR group was inferior to ACL repair group, but with no significant difference (0.05; 95% CI, −0.17 to 0.27; *p* = 0.636; *I* ^2^ = 0.0%) (Figure 3a).

### Failure rate

Failure was defined as either ACL re‐rupture or clinical findings during physical examinations, along with reported subjective instability on the surgery side. A total of five studies comprising 223 patients in the repair group and 160 in reconstruction group reported failure rates. The results showed that there was no significant difference in the failure rates between the two groups (RR, 1.10; 95% CI, 0.70–1.72; *p* = 0.695; *I* ^2^ = 27.3%) (Figure 3b).

### Publication bias

**Figure 4:**
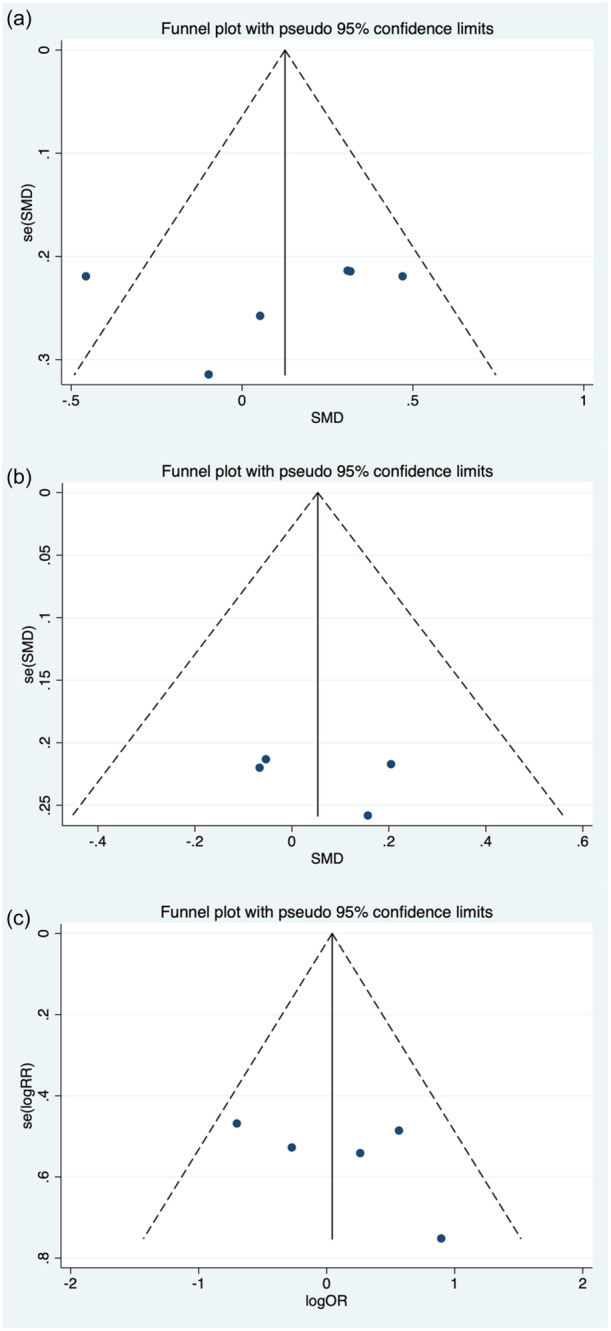
Funnel plot of meta‐analysis (a) IKDC score; (b) AP knee laxity; (c) failure rate. AP, anteroposterior; IKDC, International Knee Documentation Committee.

Due to the limited number of studies, we conducted the funnel plot and Egger test to assess publication bias in the pooling results of the IKDC score, AP knee laxity and failure rate in this meta‐analysis. As shown in Figure 4, the funnel plot indicated no obvious visual asymmetry, so no significant publication was detected. This was also statistically supported by the Egger test (*p* = 0.539, 0.581, 0.303; IKDC, AP knee laxity and failure rate, respectively).

## DISCUSSION

The most important finding of the present study was that compared with ACLR, ACL repair had similar clinical outcomes in terms of IKDC, Lysholm, Tegner score, AP knee laxity and failure rate. Thus, ACL repair could be considered as an effective alternative treatment for acute ACL injury.

In 1970s–1990s, due to the immature arthroscopic techniques and inadequate selection of the patients, the postoperative outcomes after ACL repair were unsatisfactory with high failure rates, persistent pain and instability, ACLR has become the standard treatment for ACL rupture [14]. With the development of arthroscopic technique and increasing understanding of ACL rupture, repair technique are gradually returning to attention, especially for proximal ACL tears [28]. DiFelice and List [5] showed excellent clinical outcomes with postoperative IKDC and Lysholm scores > 90, and negative Lachman test with good tissue quality at 6 years follow‐up after ACL repair. And a recent meta‐analysis (LOE IV) including nine studied using ACL repair with internal brace augmentation technique for proximal tears, reported satisfied clinical outcomes with mean IKDC scores > 87 and a 10% failure rate [33]. Besides, some LOE IV systematic reviews also indicated that ACL repair with dynamic intraligamentary stabilization or suture anchor repair for proximal tears are acceptable as there were no significant disparity results among various repair techniques [10, 13]. However, one meta‐analysis (LOE III) [19] found a higher failure rate in the repair group compared to ACLR group. As they included one study published in the last century with immature repaired technique, which reported a relatively high failure rate in the repair group. Based on the current advanced repair technique, the study should be excluded to reduce bias. In contrast, our results including 6 Level I RCTs indicated that there was no statistically significant difference between repair and reconstruction groups, although the failure rate was higher in the repair group.

As is well known, there are four histological stages during the process of ACL regeneration. During the second phase, there is significant proliferation of myofibroblast‐like cells, which potentially contributes to the retraction of the ruptured ACL and thereby hinder its repair. Thus, early repair of acute rupture before the ACL retracts can promote ACL healing, potentially achieving outcomes comparable to ACLR. However, contrary to the previous consensus, some studies have indicated that ACL repair is also applicable to chronic rupture, yielding outcomes comparable to acute ACL repair in short to midterm follow‐up [31]. In addition, age‐related indication for repair is also controversial. Some studies believed that younger patients have better healing potential after ACL repair than elders [12]. But more studies indicated that younger patients were more prone to have surgery failure following ACL repair compared to older patients [32]. As such, further studies are crucial to refining the age‐related indication for ACL repair.

It is obvious that ACL repair obviates the formation of bone tunnels, thereby preventing tunnel enlargement, which is recognized as a critical factor in hindering tendon‐bone healing after ACLR. Moreover, ACL repair eliminates the need for graft harvesting, thereby avoiding donor site morbidity, and entails no complications related to graft tendons [9].

Theoretically, ACL repair has the potential benefits over ACLR by preserving proprioception through retention of the native ACL tissue. Based on this, remnant ACLR gradually gained momentum, demonstrating satisfied clinical outcomes and a reduced graft rupture rate [2]. Some studies indicated better muscle strength recovery and improved psychological readiness after ACL repair in short‐term follow‐up compared to ACLR [3, 8]. Some studies also reported that ACL repair does not offer superior proprioception recovery compared to ACLR, as both strategies eventually enable patients to return to sports normally [34]. Due to the limited studies, proprioception‐related indication is not included in this meta‐analysis, but the advantages of ACL repair are evident. Further studies are needed to demonstrate whether ACL repair definite benefits in proprioception recovery.

### Limitation

There are several limitations in this study. First, there were only 6 studies with a small number of patients included in this meta‐analysis. We analyzed the data and found a low risk in overlap, but the generalizability still needs to be discussed. Moreover, the average age of patients was about 23.4 years, thus, the conclusions might not be suitable for an elder group. In addition, the meta‐analysis only focused on the key outcomes, did not compare the difference such as muscle strength and proprioception between repair and reconstruction group.

## CONCLUSION

ACL repair could replace ACLR as an appropriate surgical method for acute proximal ACL rupture, but the indication and selection of patients are essential to be considered. Large numbers and more high‐quality studies are still needed in the future to verify our results.

## AUTHOR CONTRIBUTIONS

**Ting Zhang**: Conceptualization; methodology; data curation; data analysis; investigation; writing original draft; preparation of revision. **Xiaojin Ye**: Conceptualization; methodology; data curation; data analysis; investigation; review and editing. **Qing Huang**: Conceptualization; methodology; data curation; data analysis; supervision; review and editing. **Ke Zhou**: Conceptualization; methodology; data curation; data analysis. **Jin Li**: Conceptualization; methodology; data curation; supervision. **Kaifeng Gan**: Conceptualization; methodology; data curation; data analysis; investigation; supervision; review and editing. All authors have read and agreed to the published version of this manuscript.

## CONFLICT OF INTEREST STATEMENT

The authors declare no conflicts of interest.

## ETHICS STATEMENT

This meta‐analysis adhered to ethical guidelines and standards established for research synthesis in the medical field. As a secondary analysis of previously published data, it did not involve direct contact with human participants or require new primary data collection. Thus, institutional review board (IRB) approval and patient consent were not applicable.

## Data Availability

Upon reasonable request, the authors can provide access to the data used for all analyses.

## References

[1] Ahmad SS , Schreiner AJ , Hirschmann MT , Schröter S , Döbele S , Ahrend MD , et al. Dynamic intraligamentary stabilization for ACL repair: a systematic review. Knee Surg Sports Traumatol Arthrosc. 2019;27(1):13–20. 30474692 10.1007/s00167-018-5301-z

[2] Allende F , Allahabadi S , Sachdev D , Gopinatth V , Saad Berreta R , LaPrade RF , et al. Comparing clinical outcomes and knee stability in remnant‐ preserving ACL reconstruction versus standard ACL reconstruction: a systematic review and meta‐analysis. Am J Sports Med. 2024;52:3635465231225984. 10.1177/03635465231225984 38551115

[3] Barnett SC , Murray MM , Badger GJ , Sanborn R , Kiapour A , Proffen B , et al. Earlier resolution of symptoms and return of function after bridge‐enhanced anterior cruciate ligament repair as compared with anterior cruciate ligament reconstruction. Orthop J Sports Med. 2021;9(11):23259671211052530. 10.1177/23259671211052530 34778483 PMC8581796

[4] Cumpston M , Li T , Page MJ , Chandler J , Welch VA , Higgins JP , et al. Updated guidance for trusted systematic reviews: a new edition of the Cochrane Handbook for Systematic Reviews of Interventions. Cochrane Database Syst Rev. 2019;10(10): Ed000142. 10.1002/14651858.Ed000142 31643080 PMC10284251

[5] DiFelice GS , van der List JP . Clinical outcomes of arthroscopic primary repair of proximal anterior cruciate ligament tears are maintained at mid‐term follow‐up. Arthroscopy. 2018;34(4):1085–1093. 29373290 10.1016/j.arthro.2017.10.028

[6] Ferretti A , Monaco E , Annibaldi A , Carrozzo A , Bruschi M , Argento G , et al. The healing potential of an acutely repaired ACL: a sequential MRI study. J Orthop Traumatol. 2020;21(1):14. 32869122 10.1186/s10195-020-00553-9 PMC7459035

[7] Gee MSM , Peterson CDR , Zhou ML , Bottoni CR . Anterior cruciate ligament repair: historical perspective, indications, techniques, and outcomes. J Am Acad Orthop Surg. 2020;28(23):963–971. 33962444 10.5435/JAAOS-D-20-00077

[8] Glasbrenner J , Raschke MJ , Kittl C , Herbst E , Peez C , Briese T , et al. Comparable instrumented knee joint laxity and patient‐reported outcomes after ACL repair with dynamic intraligamentary stabilization or ACL reconstruction: 5‐year results of a randomized controlled trial. Am J Sports Med. 2022;50(12):3256–3264. 36005281 10.1177/03635465221117777 PMC9527444

[9] Heusdens CHW . ACL repair: a game changer or will history repeat itself? A critical appraisal. J Clin Med. 2021;10(5):912. 33652689 10.3390/jcm10050912 PMC7956607

[10] Hoogeslag RAG , Brouwer RW , de Vries AJ , Boer BC , Huis In't Veld R . Efficacy of nonaugmented, static augmented, and dynamic augmented suture repair of the ruptured anterior cruciate ligament: a systematic review of the literature. Am J Sports Med. 2020;48(14):3626–3637. 32101692 10.1177/0363546520904690

[11] Hoogeslag RAG , Huis In 't Veld R , Brouwer RW , de Graaff F , Verdonschot N . Acute anterior cruciate ligament rupture: repair or reconstruction? Five‐year results of a randomized controlled clinical trial. Am J Sports Med. 2022;50(7):1779–1787. 35486517 10.1177/03635465221090527

[12] Hughes JD , Lawton CD , Nawabi DH , Pearle AD , Musahl V . Anterior cruciate ligament repair: the current status. J Bone Jt Surg. 2020;102(21):1900–1915. 10.2106/JBJS.20.00509 32932291

[13] Kandhari V , Vieira TD , Ouanezar H , Praz C , Rosenstiel N , Pioger C , et al. Clinical outcomes of arthroscopic primary anterior cruciate ligament repair: a systematic review from the scientific anterior cruciate ligament network international study group. Arthroscopy. 2020;36(2):594–612. 32014188 10.1016/j.arthro.2019.09.021

[14] Kaplan N , Wickiewicz TL , Warren RF . Primary surgical treatment of anterior cruciate ligament ruptures. A long‐term follow‐up study. Am J Sports Med. 1990;18(4):354–358. 2206080 10.1177/036354659001800404

[15] Kösters C , Glasbrenner J , Spickermann L , Kittl C , Domnick C , Herbort M , et al. Repair with dynamic intraligamentary stabilization versus primary reconstruction of acute anterior cruciate ligament tears: 2‐year results from a prospective randomized study. Am J Sports Med. 2020;48(5):1108–1116. 32125875 10.1177/0363546520905863

[16] Li J , Rothrauff B , Chen S , Zhao S , Wu Z , Chen Q , et al. Trends in anterior cruciate ligament repair: a bibliometric and visualized analysis. Orthop J Sports Med. 2022;10(10):23259671221132564. 10.1177/23259671221132564 36338352 PMC9629579

[17] Liu D , Cai ZJ , Lu WH , Pan LY , Yang YT , Li YS , et al. Eccentrically widened bone tunnels after all‐inside anterior cruciate ligament reconstruction: a computed tomography and three‐dimensional model‐based analysis. Knee Surg Sports Traumatol Arthrosc. 2023;31(6):2374–2385. 36138208 10.1007/s00167-022-07164-3 PMC10183415

[18] Malahias MA , Chytas D , Nakamura K , Raoulis V , Yokota M , Nikolaou VS . A narrative review of four different new techniques in primary anterior cruciate ligament repair: “back to the future” or another trend? Sports Med Open. 2018;4(1):37. 30094753 10.1186/s40798-018-0145-0 PMC6085215

[19] Migliorini F , Vecchio G , Eschweiler J , Schneider SM , Hildebrand F , Maffulli N . Reduced knee laxity and failure rate following anterior cruciate ligament reconstruction compared with repair for acute tears: a meta‐analysis. J Orthop Traumatol. 2023;24(1):8. 36805839 10.1186/s10195-023-00688-5 PMC9941413

[20] Montalvo AM , Schneider DK , Webster KE , Yut L , Galloway MT , Heidt RS , et al. Anterior cruciate ligament injury risk in sport: a systematic review and meta‐analysis of injury incidence by sex and sport classification. J Athl Train. 2019;54(5):472–482. 31009238 10.4085/1062-6050-407-16 PMC6602364

[21] Murray MM , Fleming BC , Badger GJ , Freiberger C , Henderson R , Barnett S , et al. Bridge‐enhanced anterior cruciate ligament repair is not inferior to autograft anterior cruciate ligament reconstruction at 2 years: results of a prospective randomized clinical trial. Am J Sports Med. 2020;48(6):1305–1315. 32298131 10.1177/0363546520913532 PMC7227128

[22] Page MJ , McKenzie JE , Bossuyt PM , Boutron I , Hoffmann TC , Mulrow CD , et al. Declaración PRISMA 2020: una guía actualizada para la publicación de revisiones sistemáticas. Rev Esp Cardiol. 2021;74(9):790–799. 34446261 10.1016/j.rec.2021.07.010

[23] Quinn M , Lemme N , Morrissey P , Fadale P , Owens BD . An update on emerging techniques and considerations in revision anterior cruciate ligament reconstruction. JBJS Reviews. 2024;12(7):e24.00047. 10.2106/JBJS.RVW.24.00047 39018384

[24] Sanders TL , Maradit Kremers H , Bryan AJ , Larson DR , Dahm DL , Levy BA , et al. Incidence of anterior cruciate ligament tears and reconstruction: a 21‐year population‐based study. Am J Sports Med. 2016;44(6):1502–1507. 26920430 10.1177/0363546516629944

[25] Sanders TL , Pareek A , Hewett TE , Levy BA , Dahm DL , Stuart MJ , et al. Long‐term rate of graft failure after ACL reconstruction: a geographic population cohort analysis. Knee Surg Sports Traumatol Arthrosc. 2017;25(1):222–228. 27522592 10.1007/s00167-016-4275-y

[26] Schliemann B , Glasbrenner J , Rosenbaum D , Lammers K , Herbort M , Domnick C , et al. Changes in gait pattern and early functional results after ACL repair are comparable to those of ACL reconstruction. Knee Surg Sports Traumatol Arthrosc. 2018;26(2):374–380. 28674740 10.1007/s00167-017-4618-3

[27] Shen Z , Chen H , Ye M , Gao Z , Li H , Lu H , et al. Early outcomes of primary repair versus reconstruction for acute anterior cruciate ligament injury: a systematic review and meta‐analysis. Medicine. 2022;101(51):e32411. 36595828 10.1097/MD.0000000000032411 PMC9794338

[28] Sherman MF , Lieber L , Bonamo JR , Podesta L , Reiter I . The long‐term followup of primary anterior cruciate ligament repair. Defining a rationale for augmentation. Am J Sports Med. 1991;19(3):243–255. 1867333 10.1177/036354659101900307

[29] van der List JP . Arthroscopic primary repair of the anterior cruciate ligament: rationale, patient selection and early outcomes (PhD Academy Award). Br J Sports Med. 2022;56:1053–1054. 10.1136/bjsports-2021-105295 35045970

[30] van der List JP , Jonkergouw A , van Noort A , Kerkhoffs GMMJ , DiFelice GS . Identifying candidates for arthroscopic primary repair of the anterior cruciate ligament: a case‐control study. Knee. 2019;26(3):619–627. 30902514 10.1016/j.knee.2019.02.004

[31] Vermeijden HD , van der List JP , DiFelice GS . Acute and delayed anterior cruciate ligament repair results in similar short to mid‐term outcomes. Knee. 2021:29142–29149. 10.1016/j.knee.2021.01.028 33626438

[32] Vermeijden HD , Yang XA , van der List JP , DiFelice GS . Role of age on success of arthroscopic primary repair of proximal anterior cruciate ligament tears. Arthroscopy. 2021;37(4):1194–1201. 33220465 10.1016/j.arthro.2020.11.024

[33] Wilson WT , Hopper GP , Banger MS , Blyth MJG , Riches PE , MacKay GM . Anterior cruciate ligament repair with internal brace augmentation: a systematic review. Knee. 2022;1:35192–35200. 10.1016/j.knee.2022.03.009 35366618

[34] Yang Y , Jin Z , Luo J , Zhang D , Shen P , Zheng D , et al. Primary repair for treating acute proximal anterior cruciate ligament tears: a histological analysis and prospective clinical trial. Front Bioeng Biotechnol. 2022;10:913900. 35711630 10.3389/fbioe.2022.913900 PMC9195517

